# Heavy Environmental Pressure in Campania and Other Italian Regions: A Short Review of Available Evidence

**DOI:** 10.3390/ijerph15010105

**Published:** 2018-01-10

**Authors:** Alfredo Mazza, Prisco Piscitelli, Andrea Falco, Maria Lucia Santoro, Manuela Colangelo, Giovanni Imbriani, Adele Idolo, Antonella De Donno, Leopoldo Iannuzzi, Annamaria Colao

**Affiliations:** 1 General Hospital Sarno, Local Health Authority ASL SA, 84087 Sarno (Salerno), Italy; 2 National Research Council (CNR), ISC, 00185 Rome, Italy; 3 Southern Italy Medical Institute (IOS), Medicina Futura Research, 80100 Naples, Italy; falco.and@gmail.com; 4 Euro Mediterranean Scientific Biomedical Institute, 72100 Brindisi, Italy; marialuciasantoro@virgilio.it (M.L.S.); manuela.colangelo95@gmail.com (M.C.); gianniimbriani@hotmail.com (G.I.); 5 Department of Biological and Environmental Sciences and Technologies (DiSTeBA), University of Salento, 73100 Lecce, Italy; adele.idolo@unisalento.it (A.I.); antonella.dedonno@unisalento.it (A.D.D.); 6 National Research Council (CNR), ISPAAM, 80100 Naples, Italy; leopoldo.iannuzzi@ispaam.cnr.it; 7 School of Medicine, University “Federico II”, 80100 Naples, Italy; colao@unina.it

**Keywords:** illegal dumping, cancer incidence, environment and health, environmental monitoring

## Abstract

The area of Naples and Campania region, in Italy, are experiencing the dramatic consequences of diffuse and illegal waste dumping, resulting in possible threats to human health. This area has been referred to as the “Land of Fires” because of the common practice of waste burning. International interest in the Campania “waste emergency” has triggered several epidemiological studies. This article is aimed at highlighting the body of evidence available concerning human and environmental contamination in the Campania region, and considers the possible lack of comparable knowledge about the situation in other areas suffering from high environmental pollution. We analyzed the results of studies addressing environmental pollution and population health in the Campania region, starting from the most recent reviews on this topic, and compared their findings with those concerning other regions. We reviewed 18 studies of epidemiological/cancer surveillance and human or animal biomonitoring. These studies show worrying results, which could be considered comparable to those available for other Italian areas impacted by heavy industrial activities. The release of environmental contaminants associated with waste incineration and waste disposal in landfills poses a risk to public health, as shown by a number of studies (although not conclusively). The current knowledge available for the Campania region is better than that available for other areas which are facing similar problems due to anthropic activities, including illegal waste trafficking. Thus, Naples and Campania could represent a valuable setting to develop general models for studies of environmental and human contamination.

## 1. Background

**Figure 1:**
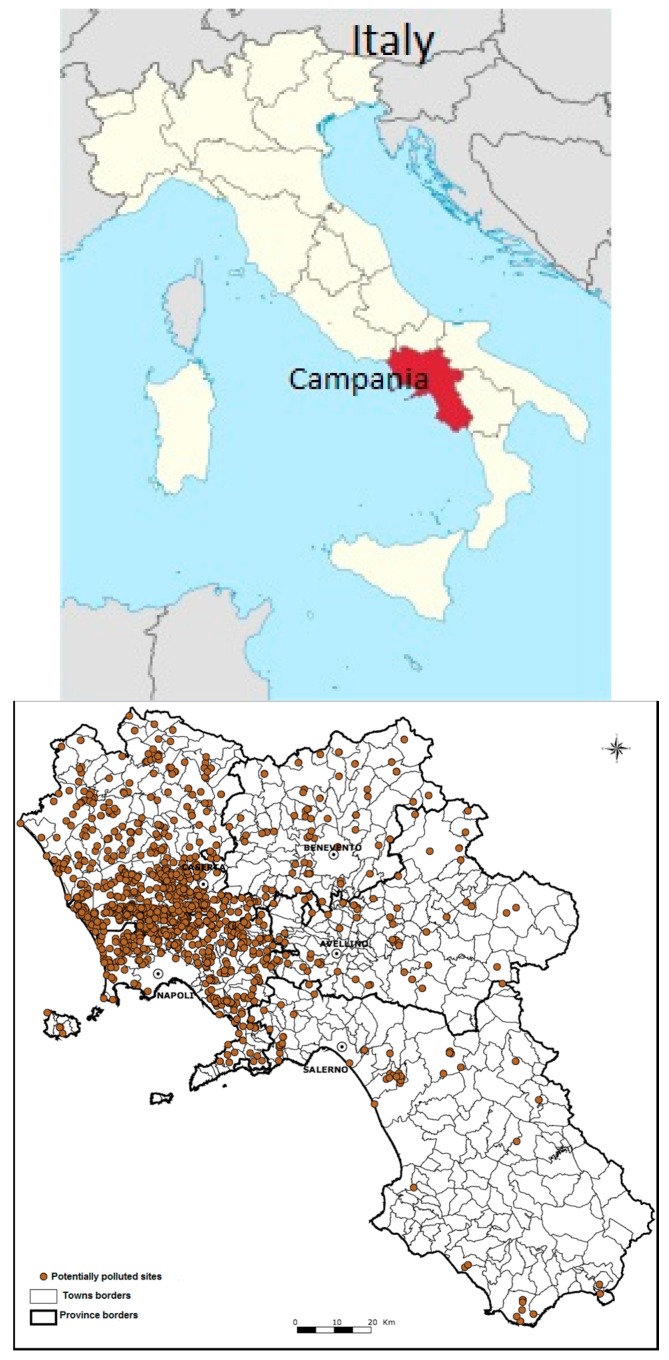
Map of illegal waste dumping sites in the region of Campania, Italy. Data from [3].

Senior and Mazza, in their publication in *The Lancet* in 2004, highlighted for the first time an increase in mortality rate due to malignancies (compared to regional and national data) in a specific area with 250,000 inhabitants in the province of Naples, the capital city of the Campania region (southern Italy), including the towns of Nola, Marigliano and Acerra, which was identified by the authors as a “triangle of death” [1]. This part of Campania has been used for illegal waste dumping by the eco-mafia for such a long time that some of this land is extremely contaminated and therefore no longer suitable for agriculture, or any other use. According to some authors, a link can be identified between the level of pollution caused by illegal dumping or the burning of waste and the high level of cancer mortality in this area [1]. The expression “triangle of death” has been widely used by the international media and it has subsequently extended to encompass all of the Campania region [2]. Due to the so-called “waste emergency” that has affected Naples and the entire Campania region in recent years, and with the spread of illegal open-air dumping and burning of urban or industrial hazardous wastes, the expression “Land of Fires” has been used to define a larger area of more than 55 towns. The “Land of Fires” refers to the entire province of Naples and the southwestern part of Caserta province; a population of approximately two million inhabitants. In Figure 1, the geographical distribution of illegal waste dumping sites identified in the Campania region, according to investigations carried out by ARPAC (Campania Regional Environmental Protection Agency) [3], is shown.

Over the past three decades, criminal organizations operating in the area (known as “Camorra”) have turned illegal waste trafficking into a prosperous business, with estimated revenues of 600 million Euros per year in Campania, alone [4,5]. The widespread incineration of waste, including plastic and rubber materials, solvents, waste from non-authorized leather factories (mainly based on Chinese undocumented workers) in such a highly populated area, has caused concern about the hazardous effects related to the uncontrolled release of dangerous pollutants into the environment, primarily dioxins, polychlorinated biphenyls (PCBs) and heavy metals [5]. The risk of environmental and biological contamination in the “Land of Fires” has been tackled by several public health interventions, some of which have required specific legislative measures. Mayors of the municipalities of the “Land of Fires”, along with several institutional authorities, including representatives of the Italian Government and the Campania region, have made a written pledge to counteract the illegal dumping of waste and to follow guidelines for their removal [5]. As a result of the surveillance activity conducted by the police since April 2014, 183 fires were reported and 1054 illegal dumping sites were identified, as at January 2016 [6]. During the same time period, the police made 56 arrests for violations of specific limitations set by Law No. 6, approved on 6 February 2014 [5]. This law also introduced systematic sampling of agricultural land potentially affected by illegal dumping and waste incineration. Furthermore, since May 2014 a monitoring program supervised by the Campania region and involving, among other institutions, ARPAC and IZSAM (Southern Italy Experimental Zooprophylactic Institute), has assessed the level of contaminants in groundwater, soil, vegetables, animal feed, animal milk and eggs in small rural farms in the “Land of Fires” [4]. A number of researchers have attempted to assess the effects of the waste crisis in certain areas of the Campania region on public health [6,7,8,9,10,11,12,13,14,15]. While judicial inquiries have unveiled the ruthless illegal waste trafficking managed by criminal organizations, biological monitoring has generated a wealth of data.

As a result of all these activities, the Campania region is actually probably the most investigated area in Italy with respect to environmental contamination of soil and vegetables. The paradox is that we now have much more information available for Campania than other Italian areas known to suffer from heavy environmental contamination (especially in northern regions). This commentary follows recent reviews [9,10] of studies carried out in the Naples area and Campania during the last decade and it is aimed at discussing the main findings of these, paying particular attention to their recommendations for public health measures that should be adopted.

## 2. Results

Eighteen papers (including two reviews) on the health effects of waste exposure and human or animal biomonitoring in Campania were reviewed. Nine papers investigated health effects, eight focused on human or animal biomonitoring and one considered both aspects. Most of these studies did not evaluate the exposure of the populations under study, the distance from legal or illegal waste sites or other confounders. Two summary tables of the studies reviewed, one for human or animal biomonitoring (Table 1) and one for health effects (Table 2), are presented to show the results.

## 3. Biomonitoring Findings

Specific biomonitoring studies [11,12,13,14,15] have assessed the levels of contaminants in humans (young breastfeeding women) living in the “Land of Fires”. Rivezzi et al. [11] measured PCDDs (polychloro-dibenzo-dioxins), PCDFs (polychloro-dibenzo-furans) and dl-PCBs (dioxin-like polychlorobiphenyls) in individual milk samples from 94 young breastfeeding women (aged 19–32) living in the Naples and Caserta provinces, according to the WHO (World Health Organization) standardized protocol. They found that all milk samples were contaminated by dioxins and dl-PCBs with an average level of dioxins of 16.6 pg toxic equivalent (TEQ)/g of fat (ranging from 7.5 to 43 pg TEQ/g of fat). Dioxin concentration in breast milk significantly increased with age (*p* < 0.01) due to a well-known bioaccumulation phenomenon. This information clearly indicated that the average contamination in the study area of Caserta and Naples is similar to that of the cities of Milan and Piacenza. However, the highly contaminated municipalities in Caserta and Naples have dioxin concentrations that are up to 1.5 times the maximum contamination recorded in Milan by Ulaszewska et al. [12]. This latter study also showed that total levels of PCDDs, PCDFs and dl-PCBs in the milk of mothers living in a small town near Naples (Giugliano) were significantly lower than those observed in controls in Milan and Piacenza [12]. A significant increase in dioxin concentration in those subjects with more exposure to the burning of waste (*p* < 0.05) was found by Rivezzi et al. [11]. Notably, Giovannini et al. observed an optimal overlapping between dioxin levels detected in these 94 breastfeeding women and those of buffalo milk [13]. On the contrary, by using a pool-sampled methodology in smaller study populations, without individual characterization of measured PCDDs, PCDFs and dl-PCBs concentrations in human breast milk, Esposito et al. [15] did not report any differences in serum levels of these pollutants when analyzing 25 subjects residing in the same “triangle of death” area identified by Senior and Mazza [1], and 33 subjects living in a different area of the Naples province (which included Pompei, Portici, Pozzuoli and Torre del Greco). In the biomonitoring SEBIOREC study (Studio epidemiologico di biomonitoraggio della Regione Campania—Epidemiological study of biomonitoring of the Campania Region) [14], 84 serum and blood samples obtained from pooled samples collected from approximately 850 donors living in 14 municipalities of the Caserta and Naples provinces and 6 milk samples from pooled samples collected from 52 women from the same areas were tested for PCDDs, PCDFs, PCBs and heavy metals including lead, mercury, arsenic, and cadmium. Three risk areas (A, B and C) associated with decreasing risk profiles were identified on the basis of the different levels of environmental pressure, primarily reflecting local activities of dumping waste, and socio-economic deprivation. Dioxin concentrations in human milk samples were found to be strongly age-dependent and positively associated with the risk area where mothers were living, while biomarker concentrations in serum and blood samples were found to be compatible with their current values in European countries and in Italy. 

Other biological organisms have been investigated in the same area for the presence of environmental pollutants. Basile et al. [16] analyzed mosses collected in the town of Acerra, and found a number of ultrastructural abnormalities, including cytoplasm vesicles and concentric multilamellar/multivesicular bodies, possibly as a result of an adaptive mechanism to environmental heavy metal pollution. In a sample of 200 frogs collected from polluted areas and uncontaminated sites in the Campania region, Maselli et al. found that frogs from a northern area of Campania located in the “Land of Fires”—and in particular those sampled near waste dumping sites—exhibited higher levels of DNA damage, as detected by using an alkaline single-cell gel electrophoresis assay [17]. In another study involving two groups of 25 river buffalo cows from the Caserta and Salerno provinces of Campania, higher mean values of dioxins (PCDDs, PCDFs) and dl-PCBs were reported along with a higher chromosome fragility in the Caserta samples compared to those collected in the Salerno area [18]. It is noteworthy that the analysis of the milk of sheep and cows bred in farms located in the north-western area of Campania showed that about two thirds of the samples assessed had dioxin level >3.0 pg TEQ/g fat, which is the safety threshold identified by the European Commission prior to 2011 [10].

## 4. Epidemiological Findings

A few studies [1,19] investigated cause-specific mortality of the population living in critically polluted areas of the “Land of Fires”. Altavista et al. [19] compared cause-specific mortality rates with respect to those expected on the basis of regional data in three towns located nearby Naples (Giugliano, Qualiano and Villaricca, a total of 150,000 inhabitants). Significantly increased cancer standardized mortality ratios (SMRs) were found in the population of Giugliano (SMR = 107.23 in males, SMR = 111.08 in females), while a non-significant increase in cancer standardized mortality ratios were found in the populations of Qualiano and Villaricca. Significantly higher standardized mortality ratios were reported for lung, brain, liver and bile duct cancer (up to approximately 200%), and also for larynx cancer (up to approximately 350%) and pleural carcinoma (up to approximately 450%). Standardized mortality ratios for circulatory diseases, diabetes and Alzheimer’s disease were also significantly increased up to approximately 250%. In the “triangle of death” area, an increase in standardized death rate (vs. the regional expected rate) for all cancers (SDR = 321.7 vs. 305.6 per 100,000 males) was reported, along with increases in standardized mortality rates for specific tumors such as liver, larynx, bladder, colorectal cancer and leukemia/lymphoma, especially in the male population [10]. In the study published by Comba et al. [20], which compared (to average regional data) cancer mortality data in a population of approximately 4 million citizens living in 196 municipalities in the provinces of Naples and Caserta, a statistically significant excess of cancer mortality was found in the provinces of Naples (+8.7% in men and +9.2% in women) and Caserta (+2.3%, but only in males). Each town was classified on a scale of 1 to 5, with class 5 corresponding to high environmental pressure, and excess of mortality for cancer.

In particular, significant excesses of liver, lung, bladder, stomach, non-Hodgkin lymphoma and kidney cancer were reported. Such an excess of cancer mortality was reported to be associated with measures of environmental contamination after adjustment for social and economic factors in a separate analysis of the same data [21]. Environmental contamination was assessed at a municipal level by computing a waste exposure index on the basis of data for legal waste landfills and illegal dumping sites. Statistically significant, excess relative risks in high-index (exposed) compared with low-index (unexposed) municipalities were found for all-cause mortality (both sexes), for all cancers (both sexes), and stomach and lung cancer (in men). Clusters of significant excess of mortality for gastric, liver, kidney lung, and bladder cancers and congenital malformations were also identified within this area [21]. A separate study investigated clusters of significant increases in the incidence of cancer [22] in neighboring municipalities. In an area including 35 municipalities and approximately half a million inhabitants, deprivation index-adjusted clusters of increased cancer incidence during the years 1997–2005 were detected in the total population, for liver and lung cancer, leukemia and soft tissue sarcomas [23].

The SENTIERI (Studio Epidemiologico Nazionale dei Territori e degli Insediamenti Esposti a Rischio da Inquinamento—National Epidemiological Study of Territories and Settlements Exposed to Pollution Risk) project, coordinated by the Istituto Superiore di Sanità from 2007–2010, assessed cancer incidence and mortality causes in 44 contaminated sites of national interest [24]. In an updated report of the epidemiological data obtained in 55 municipalities in the “Land of Fires” evaluated in the SENTIERI study, excesses of incidence, hospitalization and mortality rates were reported in the province of Naples for stomach, liver, lung, bladder, pancreatic, laryngeal, kidney, and breast cancer, and non-Hodgkin’s lymphoma. In the Caserta province, excesses of mortality and hospitalization rates were reported for stomach, liver, lung, bladder, and laryngeal cancer, and leukemia, with an excess of hospitalization for myocardial infarction in women [7,24]. The available epidemiological evidence has been systematically reviewed elsewhere, and includes a direct contribution from our study group [9,10].

## 5. Discussion

The release of environmental contaminants associated with waste incineration and waste disposal in landfills poses a risk on public health, as shown by a number of studies (although not conclusively), particularly, in the SENTIERI Study [7,24]. This is especially relevant in some areas of southern Italy that experienced a “waste emergency”, in particular, the areas of Naples and Caserta where the number of waste dumping sites in Campania has been officially estimated to exceed 6000, with 60% of these being illegal and frequently containing toxic substances [11,21]. Even under controlled conditions, waste incineration can generate and disperse acidic gases, persistent organic compounds, heavy metals and particulates into the environment [25]. As there were no waste incineration plants in the Campania region until 2009 (the year when the Acerra plant was opened), a possible correlation between these findings and the uncontrolled incineration of urban or industrial waste has been proposed [6,9,10]. However, the lack of data on exposure levels represents a major issue in the evaluation of health effects arising from exposure to illegal waste burning or disposal. Furthermore, things are made even more complicated by the difficulty in exactly identifying waste arrangements and locating illegal waste dumping sites, sinking and burning [10]. 

The concentration of dioxins and PCBs in human milk have been suggested as a reliable model for the assessment of human exposure [11,12,13,14,15]. Some data is available regarding human exposure to potentially dangerous pollutants in this area. In a review article analyzing 14 studies assessing the relationship between congenital anomalies and waste incineration, weak associations were identified for heart and neural tube defects, while stronger associations were reported for urinary tract defects and facial clefts [26]. As the authors of these studies point out, the strength of these results is limited due to the lack of biomonitoring data and reliance on distance as the only measure of exposure, as well as their inadequate statistical power due to potential confounders not being properly accounted for. Another review study including 29 papers on landfills and 31 papers on incinerators, found a relationship between an increased risk of congenital anomalies and hospitalization due to respiratory disease in people dwelling in the proximity of special waste landfills [27]. Moreover, the effect of environmental pollutants (i.e., dioxins) on human health cannot be assessed only in terms of increased cancer incidence and mortality [28]. The updating of the SENTIERI study, which presented preliminary results at the end of 2015, has raised concerns and different opinions between experts because the higher cancer incidence and mortality rates reported in the 55 towns included in the “Land of Fires” are comparable to data available for other northern and southern Italian areas strongly impacted by industrial or other types of pollution.

Some evidence is available for other southern Italian regions such as Apulia, concerning the incidence and mortality due to cancer [29,30], as well as studies on environmental contamination for the industrial area of Taranto [31,32]. Limited evidence is available concerning human contamination by persistent organic pollutants [33]. In Sicily and Sardinia, the SENTIERI study group hypothesized the role of emissions from refineries and petrochemical plants in the observed increases in mortality from lung cancer and respiratory diseases in Gela and Porto Torres [34,35]. The same authors suggested the role of emissions from metal industries in explaining the increased mortality from respiratory diseases in Taranto and in Sulcis-Iglesiente-Guspinese, and the etiological role of air pollution was proposed to explain the increase in congenital anomalies and perinatal disorders in Falconara Marittima, Massa-Carrara, Milazzo and Porto Torres [34,35]. The causal role of heavy metals (arsenic, lead, mercury), polycyclic aromatic hydrocarbons (PAHs) and halogenated compounds was suspected for the observed excesses of mortality from renal failure (in Massa Carrara, Piombino, Orbetello, Chienti and Sulcis-Iglesiente-Guspinese), and for the increases in neurological diseases (in Trento, Grado and Marano Lagoons), whilst the increase in non-Hodgkin lymphomas reported in Brescia was associated with widespread PCB pollution [34,35].

Few studies have addressed the impact on the environment and on human health of industrial areas in southeastern Sicily (Augusta/Priolo) and other chemical industries located in Ravenna and Tuscany (Rosignano) [36,37]. However, a huge body of literature on environmental pollution assessment is available for the industrial district of Porto Marghera (Venice Lagoon) [38,39,40,41].

Special attention should be paid to the situation of the Po Valley in northern Italy (including the Piemonte and Lombardia regions with the large urban areas of Turin and Milan), which can be considered as a hot spot for air pollution. This is because of the variety of emission sources resulting from intense anthropic (city traffic), industrial, agricultural and trading activities, and is also due to the orography of the Po Valley (surrounded on three sides by high mountain chains, which enhance the accumulation of particulate and pollutants). Thanks to the efforts of a study group coordinated by Forastiere and colleagues, good knowledge of air pollution and its consequences on human health are available for the Po Valley in northern Italy (with cancer registries covering a significant proportion of the population living in those areas, thus adding to environmental data and providing some reliable information on cancer incidence and mortality) as well as for the Rome and Lazio areas [42,43,44,45,46,47]. However, little evidence has been produced on water and soil contamination from the massive use of pesticides in intensive agriculture carried out in northern Italy [48].

## 6. Conclusions

In addition to the typical fall-out from anthropic activities which characterize developed countries, the Naples area and Campania region are experiencing the dramatic consequences of illegal, diffuse waste dumping and burning (including industrial disposal from northern Italy and Europe). International interest in this “waste emergency”, which have been reported in scientific papers and the mass media and referred to the “triangle of death” or “Land of Fires”, has triggered several epidemiological studies carried out by the Italian National Institute of Health (as a specific part of the SENTIERI study) and independent researchers, as well as environmental monitoring programs supported by regional authorities (ARPAC), national government (who made available a first tranche of 25 million Euros for that purpose), and EU-funded Life Plus projects carried out by Universities or National Research Council and Zooprophylactic Institutes located in Campania. According to all these research activities, a small proportion of soils in Campania are contaminated (less than 13%), and in these, the pollutants most frequently found were dioxins, PAHs and some heavy metals. Finally, no significant contamination has been found in vegetables [49]. On the other hand, epidemiological cancer surveillance and air quality monitoring show results which could be considered comparable to those available for other Italian areas impacted by heavy environmental contamination [34,35]. Despite this, only Naples and Campania are deemed worldwide as heavily contaminated areas, thus resulting in huge economic damage. According to scientific literature and ongoing institutional research programs, the current knowledge available for the Campania region, in terms of environmental monitoring is better than that available for other areas which are facing similar problems due to anthropic activities, including illegal waste disposal. Thus, Naples and Campania could represent a valuable setting to develop general models for studies on environmental/human contamination or the alteration of metabolic/reproductive function, as well as an ideal scenario for “search and re-apply” strategies aimed at environmental restoration and transfer of knowledge or sharing of best practices in carrying out epidemiological studies on human health in polluted areas.

## Figures and Tables

**Table 1:** Summary table of studies on human or animal biomonitoring.

Authors	Donors	Biomarkers Studied	Reported Findings
Rivezzi et al. [11]	94 young breastfeeding women (aged 19–32) living in the Naples and Caserta provinces according to the WHO standardized protocol	PCDDs, PCDFs and dl-PCBs in individual milk samples	All milk samples were contaminated by dioxins and dl-PCBs with an average level of dioxins of 16.6 pg TEQ/g of fat. Dioxins concentration in breast milk significantly increased with age
Ulaszewska et al. [12]	59 healthy mothers (21 mothers from Giugliano, 22 from Piacenza and 16 from Milan)	PCDDs, PCDFs and dl-PCBs in individual milk samples	Total levels of PCDDs, PCDFs and dl-PCBs in milk samples collected in Giugliano were significantly lower than those observed in controls of Milan and Piacenza
Giovannini et al. [13]	94 young breastfeeding women (aged 19–32) living in the Naples and Caserta provinces according to the WHO standardized protocol	PCDDs, PCDFs and dl-PCBs in individual milk samples	Overlapping between dioxin levels detected in these 94 breastfeeding women and those of buffalo milk was observed
Esposito et al. [15]	25 subjects residing in the “Triangle of Death” area and 33 control subjects living in a different area of the Naples province	Serum PCDDs, PCDFs and dl-PCBs	No difference in serum levels of these pollutants between 25 subjects residing in the “Triangle of Death” area and 33 control subjects was observed
De Felip et al. [14]	For serum and blood samples: 859 subjects living in Naples and Caserta; for milk samples: 52 women from same areas	PCDDs, PCDFs, dl-PCBs, As, Hg, Cd and Pb in blood, serum and in breast milk	Biomarkers’ concentrations in serum and blood samples were found to be compatible with their current values in European countries. Dioxin concentrations in human milk samples were found to be strongly age-dependent and positively associated with the risk area where mothers were living
Basile et al. [16]	Mosses collected in the town of Acerra	Ultrastructural abnormalities	Ultrastructural abnormalities, including cytoplasm vesicles and concentric multilamellar/multivesicular bodies, were observed
Maselli et al. [17]	200 frogs collected from polluted areas and uncontaminated sites of the Campania Region	DNA damage	Frogs collected from polluted areas exhibited higher levels of DNA damage
Genualdo et al. [18]	Two groups of 25 river buffalo cows, respectively from the Caserta and Salerno provinces of Campania	PCDDs, PCDFs and dl-PCBs; chromosome fragility	Higher mean values of PCDDs, PCDFs and dl-PCBs were reported along with a higher chromosome fragility in the Caserta samples compared those ones collected in Salerno area
Triassi et al. (review) [10]	Milk samples of sheep and cows bred in farms located in the north-western area of Campania	PCDDs	About two thirds of the samples assessed had dioxin level >3.0 pg TEQ/g fat, that is the safety threshold identified by the European Commission before 2011

WHO: World Health Organization; PCDDs: polychloro-dibenzo-dioxins; PCDFs: polychloro-dibenzo-furans; dl-PCBs: dioxin-like polychlorobiphenyls; TEQ: Toxic Equivalent.

**Table 2:** Summary table of studies on health effects.

Authors	Study Subjects (Years of Observation)	Health Outcames	Reported Findings
Senior and Mazza [1]	250,000 residents in “Triangle of Death” area (2002)	Cancer mortality	All cancers SDR * = 321.7 (M) vs. regional rate 305.6 Liver SDR = 35.9 (M) vs. regional rate 15.0 Larynx SDR = 12.8 (M) vs. regional rate 8.7 Bladder SDR = 29.3 (M) vs. regional rate 21.7 Colorectal SDR = 29 (F) vs. regional rate 26.4 Leukemia and lymphoma SDR = 28.2 vs. regional rate 17.9 (M); 18.7 vs. regional rate 16.1 (F)
Altavista et al. [19]	150,000 residents in 3 municipalities of Naples (Giugliano, Qualiano and Villaricca) (1986–2000)	Cancer mortality and congenital malformations	All cancers SMR = 107.23 (M) − 111.08 (F) Liver SMR = 181.13 (F) Lung SMR = 121.85 (M) − 176.94 (F) Larynx SMR = 211.85 (M) Bladder SMR = 130.12 (M) Stomach SMR = 56.1 (M)
Triassi et al. (review) [10]	250,000 residents in “Triangle of Death” area (2002)	Cancer mortality	In the “Triangle of Death” area, an increased in standardized death rate (vs. regional expected rate) for all cancers (SDR = 321.7 vs. 305.6 per 100,000 males) was reported, along with increases in standardized mortality rates for specific tumors such as liver, larynx, bladder, colorectal cancer and leukemia/lymphoma, especially in male population
Comba et al. [20]	About 4 million residents in 196 municipalities of Caserta and Naples (1994–2001)	Cancer mortality and congenital malformations	A statistically significant excess of cancer mortality was found in the provinces of Naples (+8.7% in men and +9.2% in women) and Caserta (+2.3% but only in males). In particular, significant excesses of liver, lung, bladder, stomach, non-Hodgkin lymphoma and kidney cancer were reported. Malformations: total, cardiovascular and urogenital
Martuzzi et al. [21]	About 4 million residents in 196 municipalities of Caserta and Naples (1994–2001)	Cancer mortality and congenital malformations	An excess of cancer mortality was reported to be associated with measures of environmental contamination. Statistically significant excess relative risks in high-index (exposed) compared with low-index (unexposed) municipalities were found for all-cause mortality (both sexes), for all cancers (both sexes), and stomach and lung cancer (in men)
Fazzo et al. [22]	About 4 million residents in 196 municipalities of Caserta and Naples (1994–2001)	Cancer incidence and congenital malformations	Clusters of significant increases of cancer incidence were detected for liver, lung, bladder, stomach and kidney cancer. Malformations: total, cardiovascular, urogenital and limb
Fazzo et al. [23]	About 5 million residents in 35 municipalities of Naples Province (1997–2005)	Cancer incidence and congenital malformations	Clusters of increased cancer incidence during the years 1997–2005 were detected in the total population for liver and lung cancer, leukemia and soft tissue sarcomas
Comba et al. [24]	Residents in 55 municipalities of the “Land of Fires” (2007–2010)	Cancer incidence and mortality causes	Excesses of incidence, hospitalization and mortality rates were reported in the Province of Naples for stomach, liver, lung, bladder, pancreatic, laryngeal, kidney, breast cancer, and non-Hodgkin’s lymphoma.
Pirastu et al. [7]	Residents in 55 municipalities of the “Land of Fires” (2007–2010)	Cancer incidence and mortality causes	In the Caserta province, excesses of mortality and hospitalization rates were reported for stomach, liver, lung, bladder, laryngeal cancer and leukemia.
Barba et al. (review) [9]	Residents in polluted areas of the Campania region	Cancer mortality and congenital malformations	Significant increase in cancer mortality and malformation occurrence in specific areas of the Campania region, where improper waste management and illegal waste trafficking have been repeatedly documented, was reported

M = male; F = female. SDR: standardized death rate; * Per 100,000 population.
